# *Salmonella enterica* subspecies *enterica* serovar Choleraesuis in a German wild boar population: occurrence and characterisation

**DOI:** 10.1186/s13028-018-0422-4

**Published:** 2018-10-29

**Authors:** Ulrich Methner, Sabine Merbach, Martin Peters

**Affiliations:** 1 Institute of Bacterial Infections and Zoonoses at the Friedrich-Loeffler-Institute, Federal Research Institute for Animal Health, Naumburger Str. 96a, 07743 Jena, Germany; 2 Chemisches und Veterinäruntersuchungsamt Westfalen, Zur Taubeneiche 10-12, 59821 Arnsberg, Germany

**Keywords:** Epidemiology, Germany, *Salmonella* Choleraesuis, Typing, Wild boar

## Abstract

**Background:**

The swine-adapted serovar Choleraesuis of *Salmonella enterica* subspecies *enterica* is found rarely in domestic pigs in Germany. However, a considerable and increasing number of *S.* Choleraesuis organisms have been isolated from wild boars in Germany in recent years. To investigate a possible epidemiological context, *S.* Choleraesuis strains from a regional German wild boar population and other hosts were characterised by genotyping methods.

**Results:**

Macrorestriction analysis, biochemical differentiation and antimicrobial susceptibility typing enabled the identification of several clusters of *S.* Choleraesuis. Some clusters occurred almost permanently in a certain district, whereas other groups circulated among different wild boar herds in larger regions. Non-porcine hosts were infected with the same cluster as the wild boars.

**Conclusions:**

The emergence of *S.* Choleraesuis in wild boars might be caused by a higher prevalence in the wild boar population, but also the higher awareness to infections with African swine fever may have resulted in a higher number of examined animals. Separation of wild boar populations and, as a result, also the diverse *S.* Choleraesuis organisms might be due to natural barriers and artificial barriers like arterial roads. The findings of *S.* Choleraesuis in domestic pigs emphasize the importance of strict biosecurity measures to prevent transmission from wild boars of this but also other pathogens. To avoid risks for humans by zoonotic pathogens regular inspections of meat from wildlife need to be conducted.

## Background

Pigs might be infected with different non-host adapted *Salmonella* serovars, which rarely produce systemic infections but are able to colonise the alimentary tract and gut-associated lymphoid tissue [1]. *S.* Typhimurium and *S.* Derby are the worldwide most commonly detected non-typhoid serovars in pigs, however, all *Salmonella* organisms isolated from pigs are considered a hazard for public health [2]. *Salmonella enterica* subspecies *enterica* serovar Choleraesuis is defined as host-adapted but not host-restricted on the basis that 99% of incidents are associated with pigs [3] and causes paratyphoid in swine with clinical manifestations of enterocolitis and septicaemia [4, 5]. However, it does naturally infect also other host species, including man, in which the disease can be severe. Although *S.* Choleraesuis is not often detected from human sources in the United States [6] and the European Union [2], it is an important serovar in several Asian countries. In Thailand [7] and Taiwan [8] *S.* Choleraesuis is not only frequently isolated from humans but identified as the main cause of salmonellosis. During 1950s and 1960s, *S.* Choleraesuis was the predominant serovar isolated from pigs worldwide [1]. Subsequently its prevalence has declined in numerous countries to a point where it was infrequently reported [9, 10]. At present time, this serovar is still prevalent in North America and Asia [11], but is rarely detected in Australia and Western European countries [12]. A baseline survey on the prevalence of *Salmonella* in slaughter pigs in the EU from 2006 to 2007 revealed that *S.* Choleraesuis was detected in 4 out of the 25 participating Member States in altogether only 10 out of 2600 lymph nodes examined [12]. In Germany, the National Reference Laboratory for *Salmonella* received from 2001 to 2008 not a single strain of *S.* Choleraesuis isolated from domestic pigs for further typing [personal communication, Szabo] indicating that the swine adapted serovar was not resident in the pig population in Germany. In contrast, *S.* Choleraesuis was isolated in regional laboratories in some federal states from wild boars. Reports on the occurrence of *S.* Choleraesuis in wild boars in Germany are infrequent and concern mainly single cases [13–16] or one epidemiological study [17]. Since 2013, the number of *S.* Choleraesuis organisms isolated from wild boars in Germany and sent to the National Reference Laboratory for *Salmonella* for further typing increased considerably from 17 in 2013 to 180 in 2017 [personal communication, Szabo], indicating a marked rise in prevalence in the German wild boar population. In a regional diagnostic laboratory in the federal state North Rhine-Westphalia a strong increase of septic salmonellosis in wild boars due to *S.* Choleraesuis was also observed during the last years. The swine adapted serovar was isolated in this region in a few cases even from domestic pigs, two foxes (*Vulpes vulpes*), a badger (*Meles meles*) and a red deer (*Cervus elaphus*). The aim of the present study was to characterise the *S.* Choleraesuis strains from the different hosts by phenotyping and genotyping methods in order to investigate a possible epidemiological connection.

## Methods

### Bacterial strains

A total of 31 strains of *S.* Choleraesuis isolated [18] from 31 wild boars of different age, i.e. shoats, piglets under 12 months, yearlings between 12 and 24 months, and adults over 24 months were included in the study. This selection of strains represents the entire spectrum of isolates found in the administrative district of Arnsberg in the German federal state North Rhine-Westphalia in 2017. Altogether 46 wild boars were examined from the same district in this period. *S.* Choleraesuis was detected in 39 animals, but strains from eight wild boars were excluded from the study as they were isolated at the same place of origin on the same day. The age of the animals was estimated based on tooth eruption as well as wear and tear of teeth of the lower jaw [19]. Additionally, five isolates of *S.* Choleraesuis from domestic pigs, two strains from foxes and one strain each from a badger and a red deer detected in 2017 or 2018 in this region were also included. Depending on the presence of gross lesions, tissue samples from affected organs (e.g. liver, spleen, lung, bone lesions, segments of the intestine, and intestinal content) were collected and examined according to the standard method [18]. All *Salmonella* isolates were serotyped using poly- and monovalent anti-O as well as anti-H sera (SIFIN) according to the Kauffmann-White scheme [20], the biochemical profile of the strains was determined to differentiate between biovars *S*. Choleraesuis sensu stricto and *S*. Choleraesuis variatio Kunzendorf [21], using the identification system API 20E (Analytical Profile Index 20 tubes test for Enterobacteriaceae, bioMerieux, Nürtingen, Germany).

### Antimicrobial susceptibility testing

Antimicrobial susceptibility of the *S.* Choleraesuis strains was assessed by determining the minimum inhibitory concentration (MIC) using the broth microdilution method with Sensititre™ EUVSEC plates (Trek Diagnostic Systems Ltd, East Grinstead, United Kingdom). Epidemiological cut-off values according to the European Committee on Antimicrobial Susceptibility Testing (EUCAST) were used [22]. Antimicrobial susceptibilities to sulfamethoxazole (SMX), trimethoprim (TMP), ciprofloxacin (CIP), tetracycline (TET), meropenem (MERO), azithromycin (AZI), nalidixic acid (NAL), cefotaxime (FOT), chloramphenicol (CHL), tigecycline (TGC), ceftazidime (TAZ), colistin (COL), ampicillin (AMP) and gentamicin (GEN) were examined.

### Genotyping using pulsed-field gel electrophoresis (PFGE)

Macrorestriction analysis was carried out as described previously [17].

### Pathomorphological and histological examination of the animals

A complete post-mortem examination was carried out by veterinarians on all wild boar carcasses submitted to the regional diagnostic laboratory in the federal state North Rhine-Westphalia by hunters or local veterinary authorities. African and Classical swine fever were ruled out by means of PCR, *Brucella* infection and Aujeszky’s disease have been excluded by serological methods. Wild boars examined were found dead or were shot by hunters because of overt clinical disease (abnormal behaviour, lost escape behaviour). Road killed animals were not submitted. Animals weighed between 1.5 and 53 kg. Depending on the state of decomposition samples of brain, lung, heart, liver, spleen, kidney, and intestine were fixed in 10% neutral buffered formalin. From animals with swollen feet, metaphyses of long bones were included. Bones were decalcified using hydrochloric acid (Osteomoll, Merck, Darmstadt, Germany). The formalin fixed tissue was further processed for histology using routine techniques. Sections were cut at 5 μm and stained with hematoxylin and eosin for microscopic examination. Domestic pigs and non-porcine game animals with *S.* Choleraesuis findings were necropsied in order to determine the cause of illness and death. Processing of samples from these animals for histology was identical as described above.

## Results

### Serological and biochemical characterisation of *S.* Choleraesuis

All isolates were typed according to the Kauffmann-White scheme and revealed the complete antigenic formula (6, 7: c: 1, 5) for *S.* Choleraesuis. All 40 *S.* Choleraesuis strains from different hosts were H_2_S positive and, therefore, could be referred to as biovar Kunzendorf (Table 1). The isolates revealed also identical fermentation patterns for sorbitol (SOR), melibiose (MEL) and arabinose (ARA). Therefore, only one “Analytical Profile Index” of the *S.* Choleraesuis isolates was identified (6 504 510: H_2_S+, SOR+, MEL−, ARA−).

**Table 1:** Characteristics of *Salmonella* Choleraesuis strains

Animal origin (number of strains)	MIC^a^ (µg/mL) to SMX	Macrorestriction pattern		Macrorestriction cluster^d^
		*Xba* I X^b^	*Spe* I S^c^	
Wild boars (n = 18)	256	1	1	A1
Domestic pigs (n = 2)	256	1	1	A1
Badger (n = 1)	256	1	1	A1
Deer (n = 1)	256	1	1	A1
Wild boars (n = 2)	> 1024	1	1	A2
Domestic pigs (n = 2)	> 1024	1	1	A2
Wild boars (n = 7)	> 1024	2	2	B2
Foxes (n = 2)	> 1024	2	2	B2
Wild boars (n = 2)	256	1	3	C1
Wild boars (n = 2)	> 1024	1	3	C2
Domestic pigs (n = 1)	256	1	4	D1

Analytical profile index: 6 504 510 (identical in all strains)

^a^MIC to sulfamethoxazole (SMX):1 = 256; 2 = > 1024 (addition to macrorestriction cluster^d^)

**Fig. 1:**
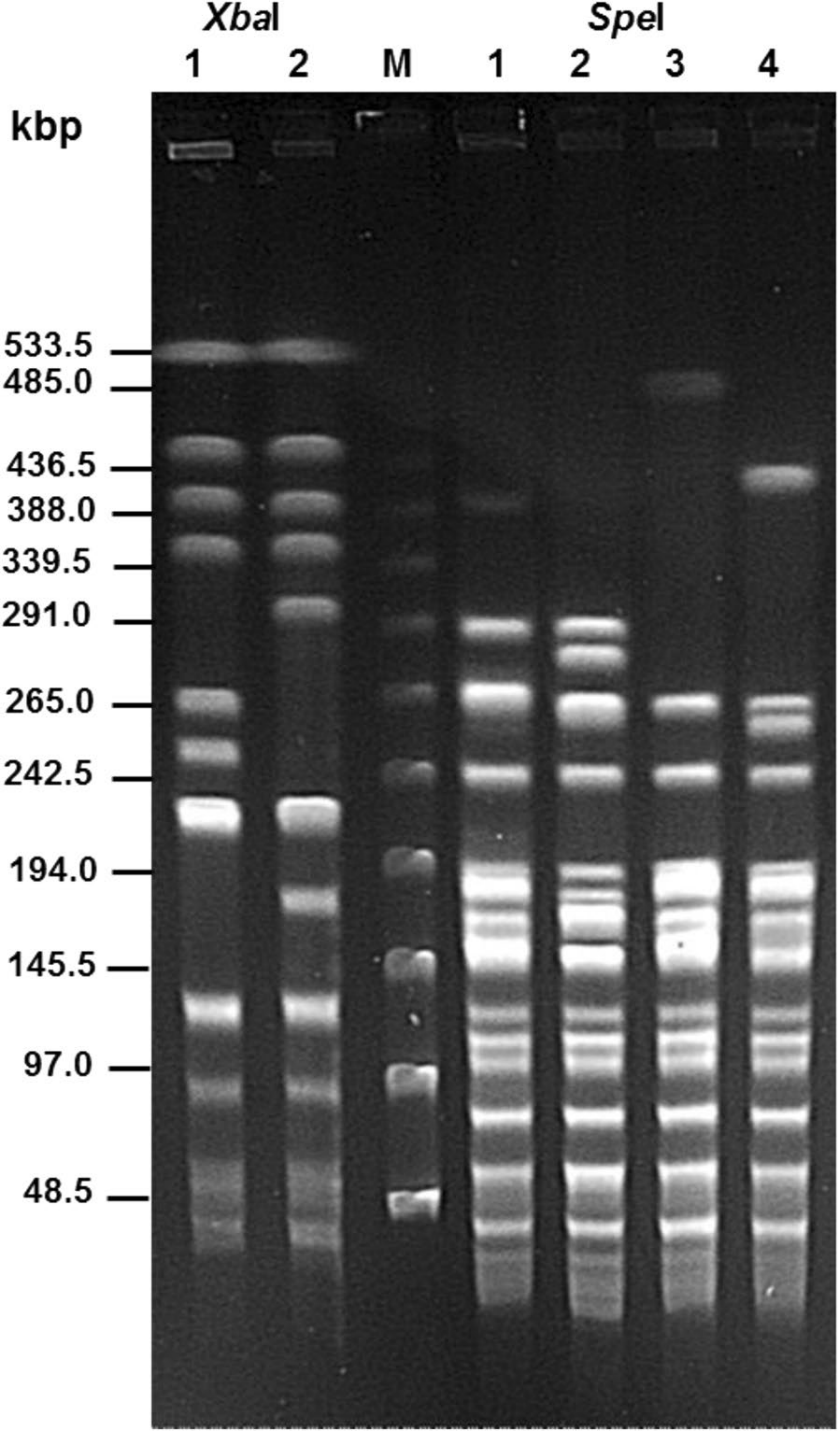
*Xba*I (X1, X2) and *Spe*I (S1, S2, S3, S4) macrorestriction patterns of 40 *Salmonella.* Choleraesuis strains from 31 wild boars, 5 domestic pigs, 2 badgers, 1 fox and 1 red deer

^b^Pattern numbers correspond to lane numbers in Fig. 1

^c^Pattern numbers correspond to lane numbers in Fig. 1

^d^Macrorestriction cluster as combination of macrorestriction patterns^b, c^ and MIC^a^

### Antimicrobial susceptibilities

All 40 *S.* Choleraesuis strains tested were sensitive to 12 out of 14 antimicrobial compounds. The organisms were all resistant to ciprofloxacin (MIC = 0.12 µg/mL). Twenty five out of the 40 strains revealed a MIC of 256 µg/mL for sulfamethoxazole and were, therefore, sensitive to this antimicrobial substance. The other 15 *S.* Choleraesuis isolates were resistant to sulfamethoxazole (MIC > 1024 µg/mL) (Table 1). This distinctive feature was also used to differentiate the *S.* Choleraesuis strains beyond the macrorestriction cluster. MIC values of 256 µg/mL were designated as 1 and MIC values of > 1024 µg/mL were termed as 2 and added to the single macrorestriction cluster to further distinguish between the *S.* Choleraesuis organisms (Table 1). Levels of susceptibility against the other antimicrobial substances did not reveal differences between the strains, the resistance pattern of the *S.* Choleraesuis strains was nearly identical. MIC values (µg/mL) of the strains were: TMP (0.5), TET (8), MERO (0.06), AZI (16), NAL (8), FOT (< 0.25), CHL (16), TGC (1–2), TAZ (1), COL (< 1), AMP (4) and GEN (2).

### Macrorestriction analysis

Two different restriction endonucleases, *Xba*I and *Spe*I (Fig. 1) were used to cleave whole-cell DNA of 40 *S.* Choleraesuis isolates originating from different regions in the district of Arnsberg in the federal state North Rhine-Westphalia (Table 1). *Xba*I digestion yielded two (X1 to X2) different patterns, *Spe*I digestion resulted in four (S1 to S4) patterns. Results of macrorestriction analysis allowed the assignment of the 40 *S.* Choleraesuis strains examined in this study to macrorestriction clusters A, B, C and D (Table 1).

### Combining of phenotyping and genotyping results

**Fig. 2:**
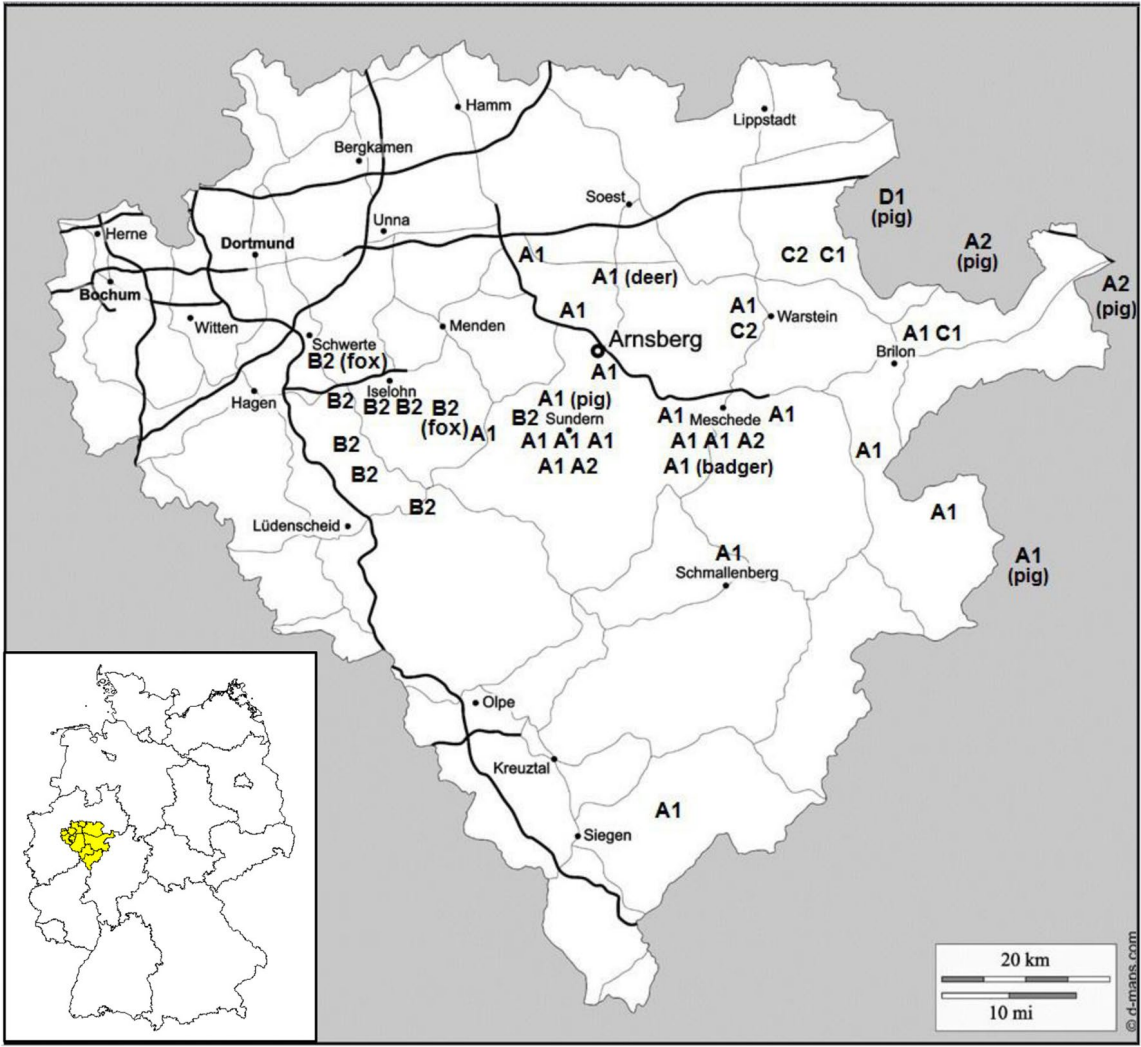
Distribution of macrorestriction clusters (A1, A2, B2, C1, C2, D1) of *Salmonella* Choleraesuis isolates from wild boars, domestic pigs, foxes, badger and red deer according to their place of origin in the administrative district of Arnsberg in the federal state North Rhine-Westphalia in Germany

The different susceptibility patterns of the *S.* Choleraesuis organisms to sulfamethoxazole enabled a further distinction of the macrorestriction clusters A, B, C and D in A1, A2, B2, C1, C2 and D1 (Table 1). The distribution of the strains according to both their affiliation to the different macrorestriction clusters and their place of origin in the district of Arnsberg in the federal state North Rhine-Westphalia are presented in Fig. 2. It could be shown that the discriminative groups are predominant in different regions. *S.* Choleraesuis strains isolated from both wild boars and foxes belonging to cluster B2 were found only in the west whereas strains of group C (C1 and C2) originated only from the east of this region. Clusters A1 and A2 were detected in the centre of the district Arnsberg, however, nearly completely separated from cluster B2. Also in this region the strains isolated from a badger, a red deer and a domestic pig did belong to the same group (A1) as the strains from wild boars. The few strains from cluster A2 were rather widely distributed in the Arnsberg district and two of them were isolated from domestic pigs. One strain isolated from a domestic pig revealed a unique macrorestriction pattern (D1), which was not detected from any other host. In total, macrorestriction cluster A1 was the most widespread type in this district, reaching even regions in the south of the Arnsberg area and still further towards the federal state of Hessen.

### Pathological findings in wild boars and other hosts

Nearly all boars (especially shoats and juveniles) examined showed signs of septicaemia. Pneumonia, congested liver with miliary necroses, enlarged spleen and hyperaemic lymph nodes were observed regularly. The animals revealed haemorrhages associated with endothelial damage and microvascular thrombosis in multiple organs like lungs, kidneys and in a few cases also in the epiglottis. Enterocolitis was not detected in any of the wild boars. Five animals were suffering from severe osteomyelitis and physitis with swollen feet. Histologically, necrotising hepatitis and splenitis as well as fibrinopurulent necrotising bronchopneumonia were the hallmarks of *S.* Choleraesuis infection in wild boars. In most cases, it was possible to cultivate pure colonies of the agent from different organs directly without pre-enrichment in buffered peptone water. The pathological findings in domestic pigs were less pronounced. One pig showed signs of septic salmonellosis with necrotising hepatosplenitis. The other domestic pigs suffered from bronchopneumonia and polyarthritis, in these animals the agent was isolated only from the intestine. From the two foxes, *S.* Choleraesuis was detected from a splenic abscess and the liver, respectively. The swine adapted *Salmonella* serovar was also isolated from liver, spleen and intestine of a badger suffering from focal cerebellar encephalomalacia, bronchopneumonia and necrotising hepatitis as well as from the intestine of a red deer. This animal originating from a game animal enclosure with public access was in poor nutritional condition, had a severe lung worm infestation, multiple abscesses in lung, lung lymph nodes and spleen, and a chronic peritonitis.

## Discussion

The occurrence of the swine-adapted serovar *S.* Choleraesuis in domestic pigs is considered as major problem for the pig industry in Asia and North America [11]. Only occasional findings are reported from Australia and Europe [12]. The recent report on zoonoses in the European Union provided no evidence on the occurrence of *S.* Choleraesuis, neither in pigs nor in humans [2]. In Germany, *S.* Choleraesuis was isolated from pigs or pork in 2014 in only two cases [23] indicating that the swine adapted serovar is not resident in the domestic pig population in this country. Reports on clinical cases or outbreaks of *S.* Choleraesuis in domestic pigs [16, 24] are also seldom. In contrast, the number of *S.* Choleraesuis isolates from wild boars sent to the German National Reference Laboratory for *Salmonella* increased from 17 in 2013 to 180 in 2017 [personal communication, Szabo], suggesting a rising prevalence in the German wild boar population. Strains of other serovars of *Salmonella* from wild boars in Germany were submitted only in a few cases for further typing. Several reasons may be responsible for this development: (i) a real increase of *S.* Choleraesuis in wild boars as result of the increased number of animals in the wild boar population [25], (ii) the higher awareness to possible African swine fever infections [26] as similarities in gross lesions with *S.* Choleraesuis infections [16] might have resulted in a higher number of animals sent to regional diagnostic laboratories, and (iii) an increase in virulence due to a possibly newly emerged clone of *S.* Choleraesuis. The diagnostic laboratory of the administrative district Arnsberg notified also an increase of septic salmonellosis due to *S.* Choleraesuis in wild boar submissions from 2006 (0 of 10) to 2017 (36 of 46). The generalised lesions in wild boars but also in other hosts in this study confirmed earlier observations and indicated the highly invasive character of this pig-adapted serovar [15–17, 24, 27]. The strong similarities in gross lesions after infection with *S.* Choleraesuis and African swine fever demand a high awareness of hunters, farmers, veterinarians and competent authorities in the control of these diseases. However, despite the actual threat by African swine fever, other diseases (classical swine fever, other septicaemic bacterial infections e.g. with *Streptococcus suis* and *Mycoplasma suis* or *Brucella suis* biovar 2) in pigs must also be considered in the differential diagnosis [28].

To detect a possible epidemiological connection between the *S*. Choleraesuis strains originating from both, different regions and hosts, PFGE as well as biochemical differentiation and antimicrobial susceptibility typing were applied to discriminate among the isolates [29, 30]. Several PFGE analyses of *S*. Choleraesuis isolates have provided reliable information on the discrimination among field isolates, between wild-type and vaccine strains, but also on the association between isolates of human and porcine origin [8, 17, 24, 29, 31]. In this study the macrorestriction analysis with two different enzymes subdivided 40 *S.* Choleraesuis isolates into 4 macrorestriction clusters A, B, C and D. The different sulfamethoxazole susceptibility patterns of the strains enabled at least partly a further differentiation of these clusters in A1/A2, B2, C1/C2 and D1. Although the biochemical characterisation of serovar *S.* Choleraesuis might be a valuable tool for the further phenotypical discrimination [17, 20, 21, 24], all strains examined in this study did belong to biovar Kunzendorf (H_2_S positive) and revealed only one “Analytical Profile Index” so that an additional distinction of the macrorestriction groups was not possible. The finding that single macrorestriction cluster occur almost permanently in only certain regions of the district Arnsberg indicates (i) that wild boars live and move only in special parts of the region or (ii) that there are barriers between regions which do not allow an exchange of the *Salmonella* organisms between different hordes of wild boars and (iii) that the *S.* Choleraesuis strains belonging to the different clusters persist and circulate in the wild boar populations in the corresponding regions. The distribution of the single cluster in this study is in great accordance with earlier results gained in another region in Germany located in a distance of about 300 kilometres [17]. Interestingly, the macrorestriction cluster of *S.* Choleraesuis strains identified in both regions did not reveal any accordance, indicating that an exchange of the organism over these long distances did not occur. The incidence of a special macrorestriction cluster in only a particular area might also be due to the amount of feed available. If there is plenty of feed the boars will stay in a territory of ca. 15 square kilometres, however, this territory may be considerably enlarged if there is a lack of feed [25]. Under these circumstances different wild boar hordes or single animals may share the same region and possibly exchange not only *S.* Choleraesuis organisms but also other pathogens. Whether this is the case in parts of the Arnsberg district is open. Also in areas with more than one macrorestriction cluster it is unknown whether these clusters occur each in one horde or in different hordes. Another factor contributing to the separation of single *S.* Choleraesuis cluster or rather the wild boar packs are with high probability barriers like motorways or other traffic routes used in high frequency. This holds true for the Arnsberg district but also other regions [17]. Therefore, it might be assumed that both natural barriers like mountains, mountain ranges or wide rivers as well as artificial barriers like arterial roads which are difficult to cross cause the separation of wild boars and, as well as their pathogens. The fact that in different non-porcine hosts like a deer, two foxes, a badger and a domestic pig the identical macrorestriction cluster was detected as in the wild boars from the corresponding region indicates, that the wild boars are the source of the infection with the swine adapted serovar *S.* Choleraesuis. Of special importance, also in view of the possible transfer of pathogens from wild boars to domestic pigs is the type of *S.* Choleraesuis cluster in domestic pigs. As wild boars or carrier wild boars might be considered as natural reservoir of *S.* Choleraesuis [11, 17], the route of transmission of this agent to the domestic pig population might be an indicator of infection routes also for other pathogens like African swine fever-virus, Classical swine fever-virus, *Brucella suis* or *Trichinella spiralis* [32, 33] and, therefore, give valuable information for the prevention of this transfer. Although the domestic pigs in this study revealed clusters of *S.* Choleraesuis identified in wild boars, it can only be suspected that wild boars are the source for the infection. Even a questionnaire in the pig farms did not yield sufficient information to finally confirm this route (data not shown). The pigs were not in free-range production and in the farms only single pigs were affected. The difficulties in analysing these routes of infection were also reported for both single cases of *S.* Choleraesuis infections [16] and large pig herds [24]. Therefore, hygiene and biosecurity measures are extremely important to prevent transmission of *Salmonella* and other pathogens from the wild boar population to domestic pigs.

The fact that *S.* Choleraesuis is the dominating serovar in the German wild boar population and that other *Salmonella* serovars are detected far less frequently [34] might indicate that wild boars are not an important host or source for foodborne salmonellosis in humans. On the contrary, studies from other countries [35–37] report on the occurrence of a wide range of *Salmonella* serovars belonging to different subspecies in wild boars. Nevertheless, because of the risk to transfer *Salmonella* and other zoonotic pathogens to humans there is a permanent need to reinforce attention on game meat inspection in order to improve the safety of pork from wild boars. Therefore, both regular inspection of meat from wildlife by official veterinarians and advice of hunters and persons who prepare and consume wild boar meat are essential measures for reducing risks to human and domestic pig health.

## Conclusions

Because of the increased emergence of *S.* Choleraesuis in the German wild boar population, the study aimed to investigate an epidemiological connection of this host-adapted serovar in wild boars and other hosts. Phenotyping and genotyping methods enabled the identification of several clusters of *S.* Choleraesuis. These groups persist either in a certain regional wild boar population or circulate in different hordes in larger areas. The separation of the wild boar hordes or the *S.* Choleraesuis strains, respectively, might be due to natural and artificial barriers like arterial roads. The detection of *S.* Choleraesuis in single cases of domestic pigs indicates the possible transmission of pathogens from wild boars and requires the establishment of an effective biosecurity system at animal farms. The inspection of meat from wildlife needs to be conducted to avoid zoonotic infections.
